# P13 Every patient who could have OPAT, should have OPAT—the importance of data

**DOI:** 10.1093/jacamr/dlac053.013

**Published:** 2022-05-31

**Authors:** Steph Williams, Hannah Bolton, Rachel Nye, Millie Watson, Alli Wood, Ann Cole

**Affiliations:** 1 York and Scarborough Teaching Hospitals NHS Foundation Trust, UK; 2 Baxter Healthcare Ltd, UK

## Abstract

**Background:**

Outpatient parenteral antimicrobial therapy (OPAT) has a clear role to play in optimizing antimicrobial stewardship and is listed as one of the Department of Health's five options for antimicrobial prescribing decision options to focus therapy. York and Scarborough Teaching Hospital NHSFT OPAT service has been operational since January 2019, treating over 438 patients and demonstrating year-on-year growth. Growth has been progressive, however a department vision established in 2021 stretched this to seek that ‘Every patient that could have OPAT, should have OPAT’—an ambitious target but not impossible with a robust evaluation of the service and a structured approach to scale.

**Objectives:**

An in-depth assessment to understand the current OPAT service and to identify pathways for further expansion together with potential efficiency and productivity gains.

**Methods:**

Working in partnership with Baxter Healthcare, a 3 month diagnostic process was undertaken with the multidisciplinary team, using a range of service and quality improvement tools to inform an in-depth assessment. A clear picture of the OPAT current state was developed through: (i) outcomes analysis; (ii) insights: pathway mapping and system flow; (iii) insights: patient experience; and (iv) insights: point of use.

**Results:**

The assessment of OPAT services provided a clear demonstration of how the service has grown from 8.4 patients per month in 2019 to 20.8 in 2020 and 27.4 in 2021, saving over 10 500 bed-days in line with the department vision and antimicrobial stewardship team goals (Figure 1). Multiple metrics have been included demonstrating clear service expansion opportunities and growth as evidenced from an initial focus on vascular, surgical and orthopaedic patients, to now include provision for patients with diabetic foot infections, bronchiectasis and intra-abdominal infections. The service has significant peaks and troughs, impacted by a number of factors impacting capacity and flow including resource, points of referral and the ability to identify patients. The OPAT Good Practice Recommendation (GPR) Assessment Tool was utilized to review overall compliance with the BSAC OPAT GPRs and to plan further improvement initiatives.

**Conclusions:**

Moving forward the focus is on predictability for sustainable growth creating a more consistent service, with multiple care pathways optimized to meet individual patient needs across North Yorkshire. The Baxter partnership has offered the Trust an opportunity to understand the service potential and meet their ambitious vision. Ultimately the goal is for 75% of patients in the OPAT service to be on a self-care pathway, thus releasing time to care.

**Figure 1.**
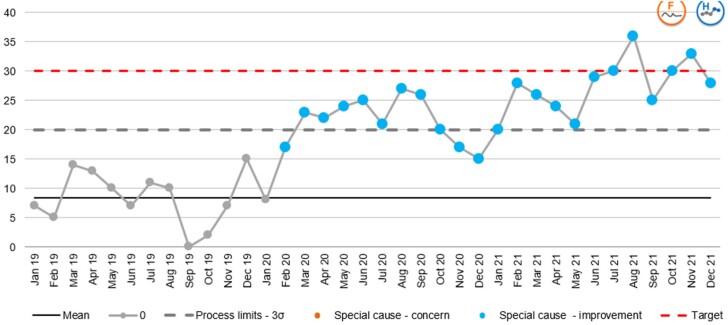
York Hospitals OPAT patient numbers, starting 1 January 2019.

